# SREBP1c mediates the effect of acetaldehyde on Cidea expression in Alcoholic fatty liver Mice

**DOI:** 10.1038/s41598-018-19466-z

**Published:** 2018-01-19

**Authors:** Qi He, Yan Diao, Tingting Zhao, Baoyu Hou, Linel Darrel Ngokana, Huan Liang, Junhui Nie, Peizhu Tan, Hui Huang, Yanze Li, Lin Qi, Yuanyuan Zhao, Ying Liu, Xu Gao, Lingyun Zhou

**Affiliations:** 1 Department of Biochemistry and Molecular Biology, Harbin Medical University, Harbin, China; 2 Translational Medicine Center of Northern China, Harbin, China; 3 Department of Clinical Laboratory, Harbin Medical UniversityCancer Hospital, Harbin, China; 4 Department of Radioimmunossay, Heilongjiang Province Hospital, Harbin, China; 5 Department of Anesthesiology, Heilongjiang Province Hospital, Harbin, China; 6 Department of Gastroenterology, Heilongjiang Province Hospital, Harbin, China; 7 Key Laboratory of Cardiovascular Medicine Research (Harbin Medical University), Ministry of Education, Harbin, China

## Abstract

Cell death inducing DNA fragmentation factor-alpha-like A (Cidea) is a member of cell death-inducing DFF45-like effector (CIDE) protein. The initial function of CIDE is the promotion of cell death and DNA fragmentation in mammalian cells. Cidea was recently reported to play critical roles in the development of hepatic steatosis. The purpose of present study is to determine the effect of chronic alcohol intake on Cidea expression in the livers of mice with alcoholic fatty liver disease. Cidea expression was significantly increased in the liver of alcohol-induced fatty liver mice. While, knockdown of Cidea caused lipid droplets numbers reduction. Next, we detected the activity of ALDH2 reduction and the concentration of serum acetaldehyde accumulation in our alcohol-induced fatty liver mice. Cidea expression was elevated in AML12 cells exposed to 100uM acetaldehyde. Interestingly, Dual-luciferase reporter gene assay showed that 100 uM acetaldehyde led to the activation of Cidea reporter gene plasmid which containing SRE element. What’s more, the knockdown of SREBP1c suppressed acetaldehyde-induced Cidea expression. Overall, our findings suggest that Cidea is highly associated with alcoholic fatty liver disease and Cidea expression is specifically induced by acetaldehyde, and this up-regulation is most likely mediated by SREBP1c.

## Introduction

Alcohol consumption is a major risk factor for many chronic disease, especially alcoholic liver disease (ALD)^1^. Alcoholic fatty liver disease is the initial stage of alcohol-induced liver disease (ALD), which is characterized by the excessive hepatic accumulation of triglycerides^2^. Alcoholic fatty liver is a worldwide health problem without effective therapeutic methods. The mechanisms by which alcohol leads to fatty liver appear to be complex, include the changes of the redox condition, transportation impairment of the synthesized lipid, inhibition of fatty acid oxidation, and the enhancement of the lipid genesis^3^. However, the currently underlying mechanisms which is on promotion of the alcoholic fatty liver is still not fully understand.

The cell death-inducing DNA fragmentation factor-alpha-like effector (CIDE) proteins contain three members (Cidea, Cideb, and Cidec) that are well known as apoptosis-inducing factors for mammalian cells^4^. However, abundant evidences indicate that CIDE play important roles in hepatic lipid metabolism^5^. Cidea is a lipid-coated protein involved in lipid droplet formation and storage that are expressed in an inducible manner^6–9^. In pathological conditions, Cidea is highly expressed in the liver of mice with hepatic steatosis fed a high fat diet (HFD)^8–11^, dystrophic mice with fatty livers^12^, obese mice or humans^8,13^ and mice with type 2 diabetes^14^. Hepatic overexpression of Cidea increases lipid accumulation and lipid droplets formation^8,9^. In contrast, Cidea^−/−^ mice exhibit reduced hepatic lipid accumulation, and knockdown of Cidea in the livers of obese mice decreases hepatic triglyceride levels and lipid droplets formation^8^. Thus, Cidea plays critical roles in promoting hepatic lipid accumulation and in the development of hepatic steatosis. However, the molecular mechanism regulating Cidea expression in the development of alcoholic fatty liver disease remains unclear.

Acetaldehyde, as a key toxin involved in alcohol-induced liver injury, increases triglycerides accumulation in recombinant HepG2 cells^15^, enhances SREBP1c expression^16,17^ and may impair the ability of PPARα to promote hepatic fat accumulation^18^. Recent studies focus on the reduced oxidation of fatty acid and the enhancement of the do novo lipogenesis. There are two important nuclear transcriptions, peroxisome proliferator-activated receptor-α(PPARα)^19^ and sterol regulatory element-binding protein-1 (SREBP-1c)^16^, are proved to be involved in alcohol-induced fatty liver. That is to say, acetaldehyde may modulate hepatic lipid metabolism and homeostasis. However, the role of acetaldehyde to promote the development of alcoholic fatty liver is still unclear. Abundant evidences have shown that Cidea promoter regions contain sterol-regulatory elements (SRE)^9,20^, the expression of Cidea was induced in the presence of saturated fatty acids (FAs)^8^ or insulin^20^. Additionally, Cidea promoter regions contain peroxisome proliferator response elements (PPREs) that are activated by a PPAR agonist^21^. Cidea expression is also regulated by the PPARα transcriptional coactivator-1 alpha^22^. Thus, we formulate a hypothesis that acetaldehyde may promote the development of alcoholic fatty liver, and it is mediated by regulating Cidea expression.

Here, we demonstrated that Cidea expression is markedly increased in the livers of chronic alcohol-fed mice and is correlated with the development of alcoholic fatty liver disease. Furthermore, Cidea expression is specifically induced by acetaldehyde, and this up-regulation is likely mediated by SREBP1c in hepatocytes.

## Results

### Hepatic Cidea expression increases in alcohol-induced fatty liver in mice

**Figure 1:**
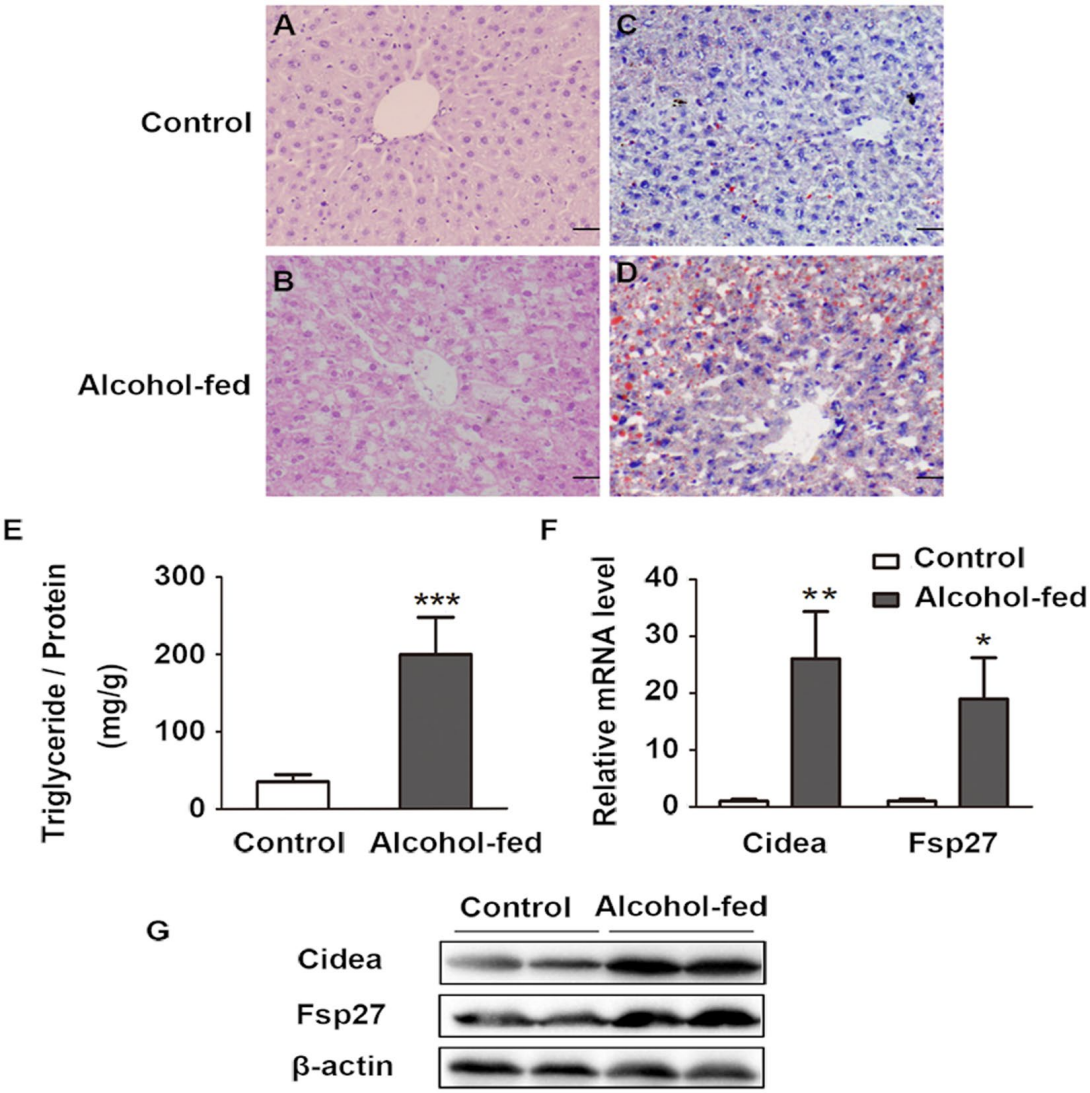
Increased hepatic Cidea expression in alcohol-induced fatty liver in mice. (**A**,**B**) Sections of the liver from control group and alcohol-fed mice were subjected to H&E staining and analysis using OLYMPUS FSX100 with a 200× objective. (**C**,**D**) Oil red O-stained liver sections (X200). (**E**) Total Triglycerides level were detected from liver tissue. Means ± SD, n = 4–5 mice per group, ***p < 0.001 vs. control group. (**F**) Relative Cidea and Fsp27 mRNA levels in control group and alcohol-fed mice. Means ± SD, n = 4–5 mice per group, **p < 0.01, *p < 0.05 vs. control group. (**G**) Cidea and Fsp27 protein levels in the liver tissue, full-length blots are presented in Supplementary Figure S1.

Recently, we have been established a new model mice of alcoholic fatty liver disease that represents a suitable model for studying the progression of AFLD^23^. As shown in Table 1, LDH, usually considered as a marker of common injury that is released during tissue damage, was significantly increased from 377.9 ± 106.62 U/L in control group to 580.9 ± 183.08 U/L in the alcohol-fed mice. ALT increased from 33 ± 6.57 U/L to 41.55 ± 9.7 U/L, and AST from 58.7 ± 11.78 U/L to 103.75 ± 61.86 U/L. Furthermore, chronic alcohol consumption increased the serum triglycerides from 0.6 ± 0.25 mg/dl in control group to 1.6 ± 0.7 mg/dl in alcohol-fed mice. HE staining and Oil Red O staining showed that hepatic lipid droplets increased in alcohol-fed mice (Fig. 1A–D). In addition, chronic alcohol consumption also significantly increased hepatic triglycerides levels (Fig. 1E). Interestingly, we found that Cidea mRNA and protein levels were markedly increased (Fig. 1F–G). Fsp27, another family member of CIDE, mRNA and protein levels were also enhanced in the alcohol-fed mice (Fig. 1F–G).

**Table 1:** Serum biochemical indexes of 12-month-old mice were subjected to chronic alcohol feeding.

	Control group (n = 10)	Alcohol-fed (n = 12)
LDH(U/L)	377.9 ± 106.62	580.9 ± 183.08**
AST(U/L)	58.7 ± 11.78	103.75 ± 61.86*
ALT(U/L)	33 ± 6.57	41.55 ± 9.7*
ALB(g/L)	31.71 ± 3.68	29.5 ± 5.76
TP(g/L)	51.21 ± 3.79	48.57 ± 5.46
GLU(mmol/L)	9.86 ± 1.97	7.09 ± 1.95**
TG(mmol/L)	0.6 ± 0.25	1.6 ± 0.7***
T-CHO(mmol/L)	2.56 ± 0.66	2.97 ± 1.24
HDL(mmol/L)	2.43 ± 0.68	2.64 ± 1.19
LDL(mmol/L)	0.29 ± 0.15	0.43 ± 0.2

LDH, lactate dehydrogenase; AST, aspartate aminotransferase; ALT, alanineaminotransferase; ALB, albumin; TP, total protein; GLU, glucose; TG, triglycerides; T-CHO, total cholesterol; HDL-C, high-density lipoprotein cholesterol; LDL-C, low density lipoprotein cholesterol.

Means ± SD. Control group, n = 10; Alcohol-fed mice, n = 12 *p < 0.05; **p < 0.01; ***p < 0.001.

### Knockdown of Cidea reduces triglyceride accumulation in alcohol-exposed cells

**Figure 2:**
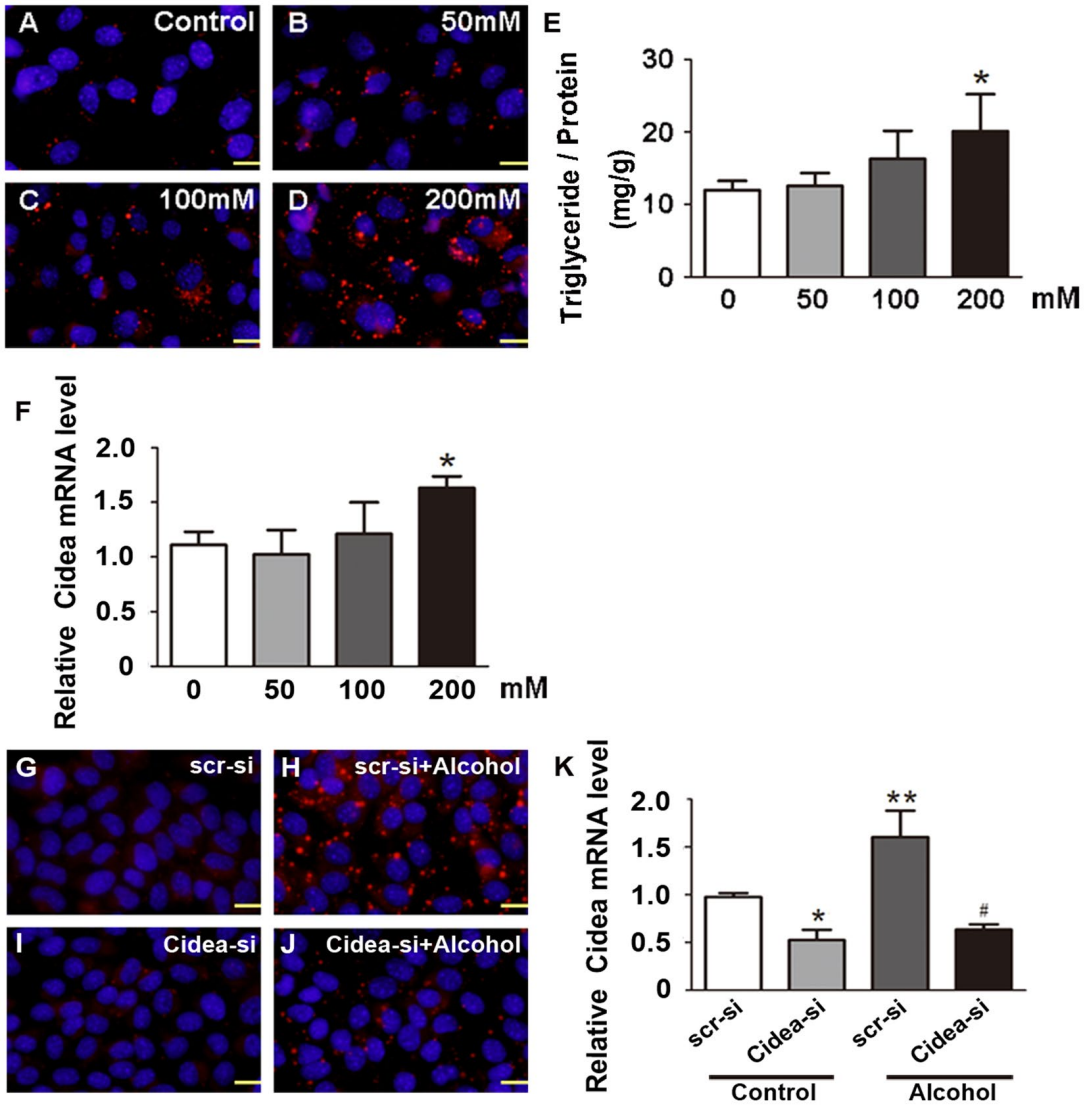
Knockdown of Cidea reduces triglyceride accumulation in alcohol-exposed cells. AML12 cells were incubated with various concentrations of alcohol for 48 hours. (**A**–**D**) Evaluation of Nile red staining (X200). The triglyceride contents (**E**) and (**F**) the Cidea mRNA levels in AML12 cells. Means ± SD, n = 3, *p < 0.05 vs. control group. (**G**–**J**) Evaluation of Nile red staining (X200) and (**K**) the Cidea mRNA levels in AML12 cells with or without Cidea knockdown by small interfering RNA (siRNA) in the presence or absence of 200 mM alcohol. Means ± SD, n = 3, *p < 0.05; **p < 0.01 vs. scr-si group; ^#^P < 0.05 vs. scr-si + Alcohol group. Scr-si, scrambled siRNA; Cidea-si, Cidea siRNA.

To evaluate the potential role of Cidea in development of alcoholic fatty liver disease, we treated AML12 cells with different alcohol concentrations. We found that alcohol increased the lipid droplets (LDs) numbers and sizes in the cells in a dose-dependent manner (Fig. 2A–D). Compared with control group, triglycerides (TG) (Fig. 2E) and Cidea expression (Fig. 2F) were increased by alcohol treatment. The LDs numbers and sizes markedly decreased in the Cidea-si + alcohol group compared with the alcohol group when Cidea was knocked down by transfecting the Cidea small interfering RNA (siRNA), at the same time, AML12 cells were treated with or without 200 mM alcohol (Fig. 2G–J). Cidea mRNA levels also decreased (Fig. 2K).

### Cidea is directly induced by acetaldehyde not by alcohol

Next, we sought to understand the molecular mechanisms of Cidea expression in hepatocytes during alcohol intake. Previous reports found that chronic alcohol feeding can cause hepatic ALDH2 dysfunction and acetaldehyde accumulation in alcoholic hepatic steatosis mice and alcohol-treated hepatic cells^24–26^. Acetaldehyde modulates hepatic lipid metabolism and homeostasis^27^.

**Figure 3:**
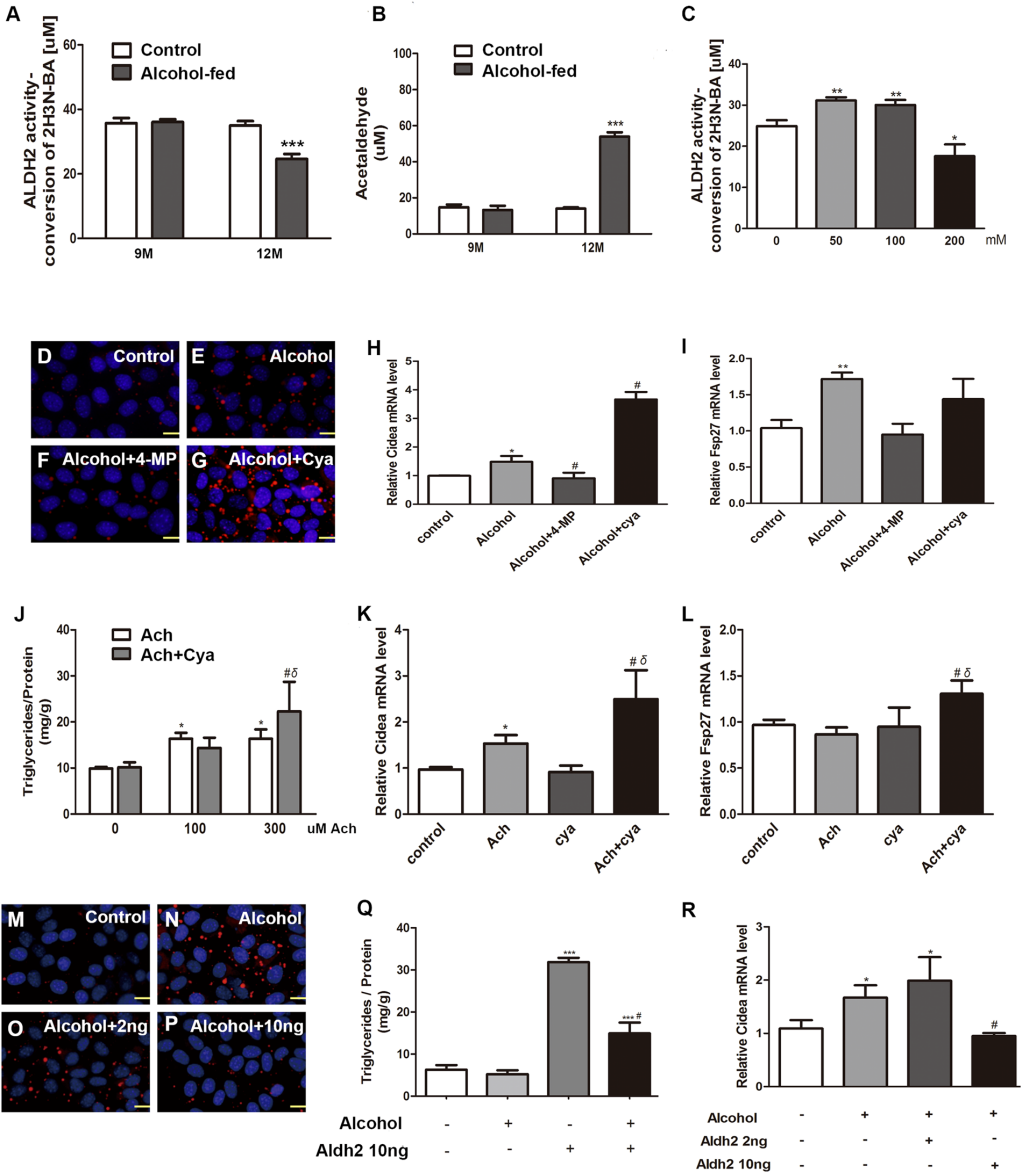
Cidea is directly induced by acetaldehyde but not by alcohol. (**A**) Activity of ALDH2 in liver tissue, ***p < 0.001 vs. control group. (**B**) Concentration of serum acetaldehyde, ***p < 0.001 vs. control group. (**C**) Activity of ALDH2 in AML12 cells were treated with different concentration alcohol, *p < 0.05; **p < 0.01. AML12 cells were treated with alcohol, 4-methylpyrazole (0.1 mM 4-MP), or cyanamide (0.1 mM Cya) for 48 h. (**D**–**G**) Nile red staining (X200). (**H**) Cidea mRNA level and (**I**) Fsp27 mRNA level were measured. Means ± SD, n = 3, *p < 0.05 vs. control group; **p < 0.01 vs. control group; ^#^P < 0.05 vs. Alcohol group; NS, not significant. AML12 cells were treated with 100 uM and 300 uM of acetaldehyde or cyanamide (0.1 mM Cya). (**J**) Triglyceride contents were measured. Means ± SD, n = 3, *p < 0.05 vs. control group; ^#^P < 0.05 vs. cya group; δ < 0.05 vs. 300 uM Ach group. AML12 cells were treated with 100 uM acetaldehyde or cyanamide (0.1 mM Cya) for 48 h. (**K**) Cidea mRNA level and (**L**) Fsp27 mRNA level were measured. Means ± SD, n = 3, *p < 0.05 vs. control group; ^#^P < 0.05 vs. cya group; δ < 0.05 vs. Ach group. AML12 cells were treated with alcohol and 2 ng or 10 ng of the Aldh2 protein for 48 h. (**M**–**P**) Nile red staining (X200) and (**Q)** Triglyceride contents and (**R**) qPCR analysis of the Cidea mRNA level were measured. Means ± SD, n = 3, *P < 0.05 vs. control group; ***P < 0.001 vs. control group; ^#^P < 0.05 vs. Alcohol group.

In our work, we assessed ALDH2 activity and acetaldehyde concentration in mice serum. Compared to control group, alcohol intake reduced ALDH2 activity in 12-month-old mice, while there was no change in 9-month-old mice (Fig. 3A). Acetaldehyde level significantly increased in alcohol-treated 12-month-old mice (Fig. 3B). Then we treated AML12 cells with different concentration of alcohol. ALDH2 activity increased in 50 mM and 100 mM alcohol, while 200 mM alcohol-exposed reduced ALDH2 activity compared with control group (Fig. 3C). Therefore, we hypothesized that the effect of alcohol on Cidea expression may directly due to its metabolic product acetaldehyde. As shown in Fig. 3D–G, 4-MP abolished the ability of alcohol to increase the LDs numbers and sizes, whereas cyanamide markedly augmented the alcohol effect. Similar to LDs numbers, Cidea mRNA level exhibited the same trend (Fig. 3H). Intriguingly, the Fsp27 mRNA level was not markedly induced by alcohol + cyanamide group compared with alcohol group (Fig. 3I). To further investigate whether acetaldehyde is directly a factor in the regulation of Cidea expression in alcoholic fatty liver disease, we treated the cells with 100 µM and 300 µM acetaldehyde with or without cyanamide. We found that triglyceride level is higher in the 300 µM acetaldehyde + cyanamide group than in 300 µM acetaldehyde group, and much higher than in control group (Fig. 3J). Next, we detected Cidea mRNA level, it increased in acetaldehyde-treated group, whereas cyanamide further enhanced the acetaldehyde effect (Fig. 3K). Interestingly, Fsp27 expression was not directly induced by acetaldehyde but Fsp27 increased in the acetaldehyde + cyanamide group (Fig. 3L). Therefore, we proposed a hypothesis that reducing acetaldehyde in alcohol metabolism could reduce Triglyceride level. We treated AML12 cells with alcohol and ALDH2 recombinant protein. Compared with the control group (Fig. 3M), the large LDs numbers significantly increased in the alcohol group (Fig. 3N); although 2 ng did not have an obvious effect (Fig. 3O), this effect was reversed by 10 ng of the ALDH2 recombinant protein (Fig. 3P), Triglycerides level increased, and the effect was reversed by 10 ng of the ALDH2 recombinant protein (Fig. 3Q). Cidea expression exhibited the same trend (Fig. 3R). These results suggest that acetaldehyde generated from alcohol metabolism is responsible for alcohol-induced Cidea expression.

### SREBP1c mediates acetaldehyde-induced Cidea expression

Acetaldehyde might be a significant factor that causes triglycerides accumulation and SREBP1c up-regulation in HepG2 cells^15–17^. In addition, Cidea promoter regions contain sterol-regulatory elements (SRE), and Cidea’s up-regulation is mediated by SREBP1c in the presence of saturated fatty acids (FAs) or insulin^9,20^.

**Figure 4:**
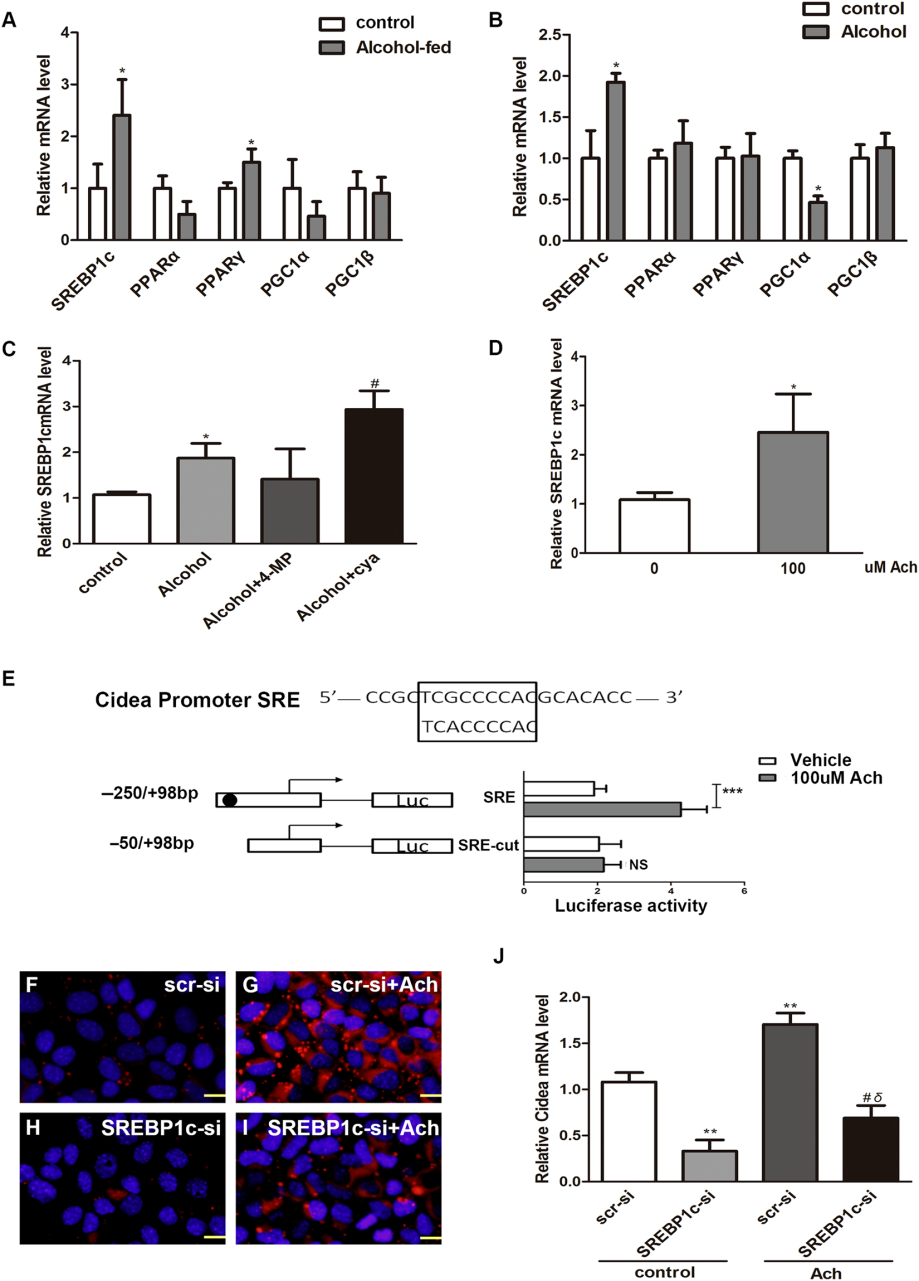
SREBP1cmediated acetaldehyde-induced Cidea expression. qPCR analysis of relative mRNA levels of key transcription factors levels in the livers of AF mice (**A**) and (**B**) Alcohol-treated AML12 cells were measured. Means ± SD, n = 4–5 mice per group, *p < 0.05; NS, not significant. (**C**) SREBP1c mRNA levels were measured in AML12 cells. Means ± SD, n = 3, *p < 0.05 vs. control group; ^#^P < 0.05 vs. Alcohol group; NS, not significant. (**D**) AML12 cells were treated with 100 uM acetaldehyde for 48 h, SREBP1c mRNA levels were measured. Means ± SD, n = 3, *p < 0.05 vs. control group. (**E**) HepG2 cells were treated with 100uM acetaldehyde for 48 h. Then Luciferase reporter plasmid were transfected into HepG2 cells with Lipofectamine 2000. Means ± SD, n = 6, ***p < 0.001; NS, not significant. (**F**–**I**) Nile red staining (X200) and (**J**) Cidea mRNA and in AML12 cells with or without SREBP1c knockdown in the presence or absence of acetaldehyde. Means ± SD, n = 3, **p < 0.01 vs. scr-si group; ^#^P < 0.05 vs. scr-si + Alcohol group; δ < 0.05 vs. SREBP1c-si group. scr-si, scrambled siRNA; SREBP1c-si, SREBP1c siRNA.

To define the specific transcription factor that mediates acetaldehyde-induced Cidea expression, we detected several key transcription factors that might regulate Cidea expression. Real-time PCR analysis of the gene levels showed a significant increase in SREBP1c and PPARα expression in the livers of AFLD mice (Fig. 4A); however, only SREBP1c expression was up-regulated in the AML12 cells (Fig. 4B). Then 4-MP abolished the ability of alcohol to increase SREBP1c mRNA level, whereas cyanamide markedly augmented the alcohol effect (Fig. 4C). Furthermore, SREBP1c expression increased in the acetaldehyde-treated group (Fig. 4D). To determine whether SREBP1c proteins were bound to the Cidea SRE element, we prepared the Luciferase reporter plasmid constructs of containing Cidea SRE element or completely deleting the SRE element of Cidea promoter and transfected into HepG2 cells. Dual-luciferase reporter assay results showed that 100 µM acetaldehyde led to the considerable activation of the Cidea reporter gene plasmid containing SRE element. However, when the reporter gene plasmid was further truncated completely deleting the SRE element, the activation was almost lost (Fig. 4E). To further verify the relationship between Cidea expression and transcription factor SREPB1c. AML12 cells were transfected with SREBP1c small interfering RNA (siRNA). The LDs numbers and sizes decreased in the SREBP1c-si + acetaldehyde group compared with the acetaldehyde group (Fig. 4F–I). Cidea expression, which was down-regulated by siRNA, was slightly elevated by acetaldehyde (Fig. 4J). Our subsequent series of results revealed that SREBP1c might serve as a transcription factor for acetaldehyde-induced Cidea expression.

## Discussion

Although Cidea mRNA is not detectable in the mouse liver^21^, Cidea has highly expressed in the liver under pathological conditions, such as HFD feeding, leptin deficiency or type 2 diabetes. High fat diet (HFD) feeding leads to hepatic steatosis as a result of higher plasma lipid levels from food intake. Unsaturated fatty acids (FAs) form the basis of the high fat diet, and Cidea expression is specifically induced by saturated FAs^8^. Interestingly, Cidea expression is an example of age-dependent augmented expression in HFD-fed mice^11^. Cidea is also reported to be markedly up-regulated in ob/ob mouse livers^8,13^, and its expression is induced by insulin in type 2 diabetic mouse livers^14,20^. Our findings showed that Cidea expression was highly increased in our alcohol-fed mice and alcohol-exposed AML12 cells. High Cidea expression resulted in significantly increased hepatic lipid accumulation and large LD numbers^8,9^. In contrast, Cidea^−/−^ mice exhibited reduced hepatic lipid accumulation when fed the HFD, and knockdown of Cidea in the livers of ob/ob mice^8^ or in hepatocytes^20^ decreased hepatic triglyceride levels and LD formation. Moreover, Cidea expression was also reduced in several genetically modified animals that were resistant to hepatic steatosis^28,29^, and concomitant administration of eicosapentaenoic acid (EPA) with a high-fat diet ameliorated hepatic steatosis in mice and down-regulated Cidea expression^9^. These studies illustrate that Cidea may act as a liver marker for the appearance of hepatic steatosis. Our results suggest that Cidea knock-down leads to an obvious abrogation of alcohol-induced Cidea expression and large LD formation in AML12 cells.

Cidec (Fsp27 in mice) like Cidea, is also a lipid-coated protein involved in lipid droplet formation and storage that are expressed in an inducible manner^6–9^. Fsp27 expression markedly elevated in both HFD-fed and ob/ob mouse livers^7,30^. Furthermore, Fsp27 has been shown to be a regulator of PPARα-dependent hepatic steatosis^7^. Therefore, Fsp27 expression in the liver is correlated with the development of hepatic steatosis. Indeed, our findings showed that the Fsp27 mRNA was up-regulated in both the livers of alcohol-fed mice and alcohol-induced AML12 cells. Previous studies reported that Cidea expression was induced by saturated fatty acids (FAs), whereas Fsp27 expression was not affected by FAs but was significantly enhanced by a PPAR agonist^8^. Interestingly, our results showed that Fsp27 expression was not directly induced by acetaldehyde but the Fsp27 level was lightly increased in the acetaldehyde + cyanamide group. Acetaldehyde accumulation impaired the activity of PPARα resulted in severe abnormal lipid metabolism^18^. In addition, acetaldehyde sensitizes HepG2 cells to TNFa by impairing mGSH transport through an ER stress-mediated increase in cholesterol^17^. However, supporting the role of pharmacologic ER stress in up-regulating de novo lipogenesis, tunicamycin (TM) and thapsigargin (Tg) treatment which induce ER stress by pharmacologic reagents significantly increased accumulation of cytosolic lipid droplet formation and hepatic lipogenesis, among these lipogenesis gene including Fsp27^31^. Thus, the molecular mechanisms that regulate Cidea and Fsp27 expression in Alcoholic fatty liver disease may be different.

A recurring emergent theory in the alcohol field is that the reinforcing properties of alcohol are not produced by the alcohol molecule, but may depend upon the action of acetaldehyde within alcohol-associated disorders including alcoholic liver disease (ALD)^32^; alcoholic cardiomyopathy^33^; central nervous system (CNS)^34^; even carcinogenic^35,36^. As acetaldehyde is a toxic molecule which is a risk factor for pathogenesis of various alcohol-associated disorders including ALD. Accumulating evidence suggests that the development of liver fibrosis in alcoholics has been linked to the oxidation of alcohol to the highly reactive compound acetaldehyde^37^. Acetaldehyde is fibrogenic and induces expression of both COL1A1 and COL1A2 genes by a mechanism dependent on the generation of H_2_O_2_^38^ and by a mechanism through Ca^2+^-independent PKC activation^39^. In fact, chronic Alcohol feeding can cause hepatic ALDH2 dysfunction and acetaldehyde accumulation in alcoholic fatty liver, that is the initial stage of alcohol-induced liver injury^24^. Interestingly, the concentration of serum acetaldehyde level in our model mice also increased. However, the effect of acetaldehyde in alcoholic fatty liver is not caused much attention by people. Previous studies suggested that Cidea promoter regions contain sterol-regulatory elements (SRE), and Cidea up-regulation is mediated by SREBP1c in the presence of saturated fatty acids (FAs) or insulin^9,20^. In addition, acetaldehyde enhanced SREBP-1c gene and protein expression, resulting in increasing lipogenic enzyme gene expression^15,17^. To further verify whether the effects of acetaldehyde on Cidea expression is directly due to SREBP1c mediated, we did Dual-luciferase reporter gene experiments, the results showed that 100 uM acetaldehyde led to the considerable activation of the Cidea reporter gene plasmid containing SRE element. Therefore, we found that SREBP1c directly mediates the effect of acetaldehyde on Cidea expression in hepatocytes.

ALDH2 is the second enzyme of the major oxidative pathway of alcohol metabolism, which is perhaps the most efficient mitochondrial ALDH isozyme^24^. However, chronic alcohol exposure caused oxidative stress may reduce the activity of ALDH2^24,25^ and ALDH2 protein level^27^, this is leading to acetaldehyde accumulation significantly increase in the blood and liver^24,26^. Several researches have showed that ALDH2 overexpression transgenic mice^40^ or concomitant administration of Alda-1^24^ with a chronic alcohol feeding can ameliorate alcoholic hepatic steatosis. Therefore, removal of excessive acetaldehyde loaded in the blood appears to be a new strategy for the treatment of alcoholic fatty liver disease. Recently, the ALDH2 recombinant protein was reported to play a protective role in the reduction of vascular contraction in AngII mice aorta^41^. Therefore, we treated AML12 cells with the ALDH2 recombinant protein and we found that Cidea expression and triglyceride contents decreased in the presence of alcohol. However, we didn’t verify the effect of ALDH2 recombinant protein on mice with alcoholic fatty liver disease. Further studies will be necessary to clarify whether the ALDH2 recombinant protein might ameliorate alcoholic fatty liver disease.

## Materials and Methods

### Animals

All animal experiments were approved by the Harbin Medical Univerisity’s Animal Care and Use Committee and conducted according to the National Institutes of Health guidelines. Male C57BL/6J mice at 8-weeks of age were housed in a room with a 12 h light/dark cycle and provided free access to food and water. The experimental group mice were fed 15% alcohol water (v/v) (alcohol-fed) and the wild-type (WT) mice were fed water for 10 months^23^.

### Cell culture

Normal mouse hepatocyte cell line AML12(ATCC, CRL-2254) was cultured in DMEM/F12 medium (Gibco, Life Technologies) supplemented with 10% fetal bovine serum (FBS) (Gibco, Life Technologies), 100 ng/ml streptomycin, 63 ng/ml penicillin G, 0.1 uM dexamethasone, and insulin-transferrin-selenium (ITS; Gibco-BRL) in 37 °C humidified incubator containing 5% CO_2_.

### Transfection of siRNA

Cells were transfected with the Cidea siRNA or SREBP1c siRNA using the X-treme GENE siRNA Transfection Reagent (Invitrogen)and treated with 200 mM alcohol or 100 uM acetaldehydes six hours after transfection.

### Nile red staining

AML12 cells were stained with Nile Red solution (0.1 mg/ml) at 37 °C for 20 min, fixed with 4% formaldehyde for 10 min, and then counterstained with DAPI (1 ug/ml) for 10 min.

### Measurement of triglycerides

The hepatic levels of triglycerides were detected using the Triglyceride Colorimetric Test kit (Ann Arbor, MI). The Cells were treated as described above.

### Plasmid construction and transfection

Mouse Cidea cDNA was amplified from mouse liver tissue cDNA by polymerase chain reaction (PCR). Different deletion fragments were cloned by PCR using mouse liver cDNA as template and insert into the Nhel/Hindlll site of the pGL3basic vector. The Luciferase reporter plasmid were transfected into HepG2 cells which already have been induced by 100 uM acetaldehyde.

### Determination of ALDH2 activity

The activity of ALDH2 is isolated from mitochondria of mouse liver tissue and AML12 cells. Conversion of 2-hydroxy-3-nitrobenzaldehyde to its benzoic acid product was followed by HPLC-based analysis by its absorbance at 340 nm as previously described^29^.

### Measurement of serum acetaldehyde

Blood was drawn from the angular vein of the eyes and was subsequently incubated at room temperature for half an hour followed by centrifugation at 5000 rpm for 5 min. Samples were then analyzed by Colorimetric Aldehyde Assay Kit, Blue (Sigma-Aldrich).

### Western blotting

Liver tissues and AML12 cell protein lysates were prepared using RIPA buffer containing complete EDTA-free protease inhibitor cocktail (Roche). A total of 30–50 ng of protein was subjected to 12% SDS-PAGE and transferred to a 0.45 um PVDF membrane (Millipore, Bedford, MA, USA). The membranes were probed with antibodies to Cidea (1:1000; ab8402, abcam) and Fsp27 (1:1000; ab77115, abcam) detected using chemiluminescence reagents.

### Real-time PCR

Total RNA was prepared from liver tissues and AML12 cells using the TRIzol reagent according to the manufacturer’s protocol (Invitrogen). Total RNA was reverse transcribed into cDNA using the High-Capacity cDNA Reverse Transcription Kit (Applied Biosystems, CA, USA). Quantitative real-time PCR was performed using the SYBR Green kit with the Applied Biosystems’ 7500 Real-Time PCR System.

### Data analysis

The results are from at least three independent experiments. Data are presented as the mean ± SD. The statistical analysis was calculated using Student’s t-test. Multiple comparisons were estimated by analysis of variance (ANOVA) followed by the Newman-Keuls test. Values of P < 0.05 were considered statistically significant.

## Electronic supplementary material

Supplementary information
