# Supplementary material for: SREBP1c mediates the effect of acetaldehyde on Cidea expression in Alcoholic fatty liver Mice
Source: Sci Rep. 2018 Jan 19;8:1200. doi: 10.1038/s41598-018-19466-z (PMC5775393; doi:10.1038/s41598-018-19466-z)

SREBP1c mediates the effect of acetaldehyde on Cidea expression in Alcoholic fatty liver Mice

Authors: Qi He[1,2#], Yan Diao[1,2#], Tingting Zhao[1,2#], Baoyu Hou[1,2], Linel Darrel Ngokana[1,2], Huan Liang[1,2,3], Junhui Nie[1,2], Peizhu Tan[1,2], Hui Huang[1,2], Yanze Li[1,2], Lin Qi[4], Yuanyuan Zhao[5], Ying Liu[6], Xu Gao[1,2,7*], Lingyun Zhou[1,2,*]

[1]Department of Biochemistry and Molecular Biology, Harbin Medical University, Harbin, China. [2]Translational Medicine Center of Northern China, Harbin, China. [3]Department of Clinical Laboratory,Harbin Medical UniversityCancer Hospital, Harbin, China. [4]Department of Radioimmunossay, Heilongjiang Province Hospital, Harbin, China. [5]Department of Anesthesiology, Heilongjiang Province Hospital, Harbin, China. [6]Department of Gastroenterology, Heilongjiang Province Hospital, Harbin, China. [7]Key Laboratory of Cardiovascular Medicine Research (Harbin Medical University), Ministry of Education, Harbin,China.

[#]These authors contributed equally to this work.

*Corresponding author
E-mail: gaoxu_671227@163.com; zhoulingyun27@sina.com

Supplementary Figure S1

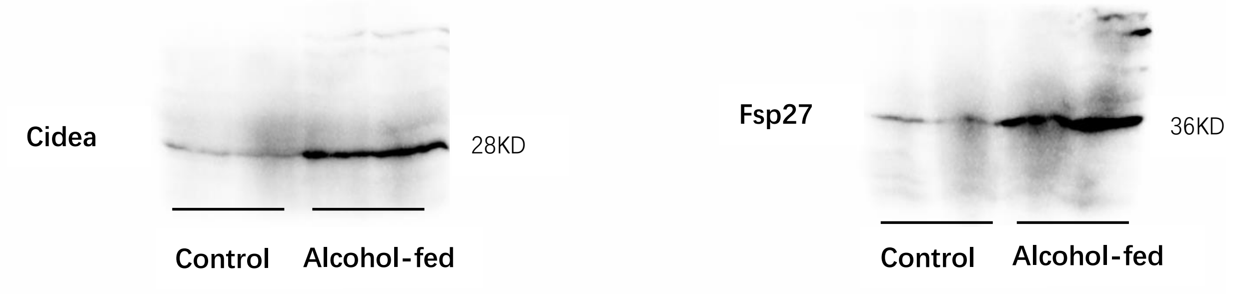

Supplement: Supplementary file 1 — Supplementary information [file 41598_2018_19466_MOESM1_ESM.pdf]
